# Proportion and Number of Upper-Extremity Musculoskeletal Disorders Attributable to the Combined Effect of Biomechanical and Psychosocial Risk Factors in a Working Population

**DOI:** 10.3390/ijerph18083858

**Published:** 2021-04-07

**Authors:** Aboubakari Nambiema, Julie Bodin, Susan Stock, Agnès Aublet-Cuvelier, Alexis Descatha, Bradley Evanoff, Yves Roquelaure

**Affiliations:** 1 Univ Angers, CHU Angers, Univ Rennes, Inserm, EHESP, Irset (Institut de Recherche en Santé, Environnement et Travail)—UMR_S 1085, F-49000 Angers, France; julie.bodin@univ-angers.fr (J.B.); alexis.descatha@inserm.fr (A.D.); yves.roquelaure@univ-angers.fr (Y.R.); 2 INSPQ—Institut National de Santé Publique du Québec, Montréal, QC H2P 1E2, Canada; susan.stock@inspq.qc.ca; 3 Department of Social & Preventive Medicine, University of Montréal, Montréal, QC H3N 1X9, Canada; 4 INRS, Département Homme au Travail, 1 rue du Morvan CS60027, 54519 Vandœuvre, France; agnes.aublet-cuvelier@inrs.fr; 5 Inserm, UMS 011, Unité Cohortes Epidémiologiques en Population, 94807 Villejuif, France; 6 CHU Angers, Poisoning Control Center, F-49000 Angers, France; 7 Division of General Medical Sciences, Washington University School of Medicine, St. Louis, MO 63310, USA; bevanoff@wustl.edu

**Keywords:** cohort study, France, upper-extremity MSD, occupational risk factor, combined effect, preventable cases, prevention

## Abstract

The objective of this paper is to assess the combined effect of occupational biomechanical and psychosocial risk factors on the incidence of work-related upper-extremity musculoskeletal disorders (UEMSDs) and estimate the proportion and number of incident cases attributable to these risk factors in a working population. Using data from the French COSALI (COhorte des SAlariés LIgériens) cohort (enrolment phase: 2002–2005; follow-up phase: 2007–2010), a complete case analysis including 1246 workers (59% men, mean age: 38 years ± 8.6 at baseline) was performed. All participants underwent a standardized clinical examination at enrolment and 1611 workers were re-examined at follow-up. Population attributable fractions and the number of UEMSD cases attributable to occupational risk factors were calculated. During follow-up, 139 UEMSD cases were diagnosed, representing an estimated 129,320 projected incident UEMSD cases in the working population. After adjusting for personal factors, in model 1, 8664 cases (6.7%) were attributable to low social support, 19,010 (14.7%) to high physical exertion, and 20,443 (15.8%) to co-exposure to both factors. In model 2, 16,294 (12.6%) cases were attributable to low social support, 6983 (5.4%) to posture with arms above shoulder level, and 5043 (3.9%) to co-exposure to both factors. Our findings suggest that many cases of UEMSD could be potentially prevented by multidimensional interventions aimed at reducing exposure to high physical exertion and improving social support at work.

## 1. Introduction

Throughout the world, upper-extremity musculoskeletal disorders (UEMSDs) (e.g., carpal tunnel syndrome and shoulder tendinopathy) are an important health problem in the working population, with a major impact on work-related disability, quality of life, and years lost due to disability [1,2,3]. In addition to their consequences on the health of workers, UEMSDs also have a serious impact on workers’ careers, absenteeism from work, and on the economic health of the companies they work for, particularly in terms of costs related to production loss, work performance, and the sustainability of workers’ activities [1,4]. In France, according to 2018 social health insurance data, the costs of all work-related MSDs (mainly UEMSDs, which comprise 91% of all MSDs) was estimated at €2 billion for companies [5].

Previous studies have provided UEMSD prevalence and/or incidence estimates. A systematic review [6] of worldwide incidence and prevalence studies in the working population showed a 12-month prevalence of UEMSDs ranging from 2.3% to 41.0%. A recent review [7] found that the annual incidence and the prevalence worldwide ranged from 0.08% to 6.3% and from 0.14% to 21.9% in the working population, respectively. Furthermore, a large amount of literature documented that UEMSDs are associated with multiple risk factors, including personal factors (e.g., age), biomechanical factors (e.g., repetitiveness of tasks), psychosocial factors (e.g., low social support), and organizational factors (e.g., machine-paced work) which commonly interact with each other in determining overall risk [8,9,10,11,12].

With regard to UEMSD prevention, occupational physicians, public health practitioners, and policy makers should prioritize interventions based on modifiable risk factors or on a combination of risk factors with the greatest effect in the working population and which prevent the greatest number of incident cases. In a previous study [13], population attributable fractions (PAFs) and the number of incident UEMSDs attributable individually to each identified risk factor were estimated for French workers in the Pays de la Loire (PdL) region. Based on the results of this previous study, this paper aims to (i) assess the combined effect of occupational biomechanical (e.g., high physical exertion) and psychosocial (e.g., low social support) risk factors on the incidence of UEMSDs among French workers in the PdL region and (ii) estimate the proportion and number of incident cases attributable to these risk factors in two prevention models in the PdL region.

## 2. Materials and Methods

Data from the French COSALI (COhorte des SAlariés LIgériens) cohort [13], which was based on data from a prospective study of MSDs and their risk factors in the working population from the French PdL region [14], were re-analyzed. Briefly, a total of 3710 workers from the French PdL region were randomly enrolled by 83 occupational physicians (OPs) (18% of OPs in the region) who had volunteered to take part in the study between 2002 and 2005. They completed a self-administered questionnaire and underwent a standardized clinical examination performed by an OP. Between 2007 and 2010, 1611 workers were re-examined by their OP. Of the 1611 workers re-examined at follow-up, a total of 365 workers with baseline UEMSDs or those with missing values for UEMSD follow-up or for at least one covariate were excluded. Complete case analyses were performed on the remining 1246 workers.

The study received approval from France’s Advisory Committee on Information Processing of Information in Health Research (“CCTIRS”) and the French Data Protection Agency (“CNIL”), initially in 2001 and again in 2006. Each worker provided written informed consent prior to enrolment.

### 2.1. Outcome Definition

Outcome was defined as incident cases of six most common clinically diagnosed UEMSDs among workers without any of the six most common clinically diagnosed UEMSDs at baseline and who met the criteria for at least one of the disorders at follow-up (based on the European consensus criteria to diagnose work-related UEMSDs for the health surveillance of epidemiologic studies) [13,15]. These UEMSDs were carpal tunnel syndrome (CTS), ulnar tunnel syndrome, De Quervain’s disease, flexor-extensor peritendinitis or tenosynovitis of the forearm-wrist region, rotator cuff syndrome (RCS), and lateral epicondylar tendinopathy (LET).

### 2.2. Covariates

Previously identified risk factors for UEMSDs [13] were assessed with a self-administered questionnaire: high perceived physical exertion at work (Borg rating perceived exertion (RPE]) scale ≥13) (yes/no), working posture with arms above shoulder level (≥2 h/day) (yes/no), low social support at work (yes/no), age (<35 years, 35–44 years, and ≥45 years), and female sex (yes/no). The high perceived physical exertion at work was evaluated using the Borg RPE scale [16], ranging from 6 (no exertion at all) to 20 (maximal exertion) and dichotomized using the threshold (Borg RPE scale ≥13) proposed by the French National Research and Safety Institute for the Prevention of Occupational Accidents and Diseases (INRS) cut-offs [17]. Working posture with arms above shoulder level (≥2 h/day) was assessed using the European consensus criteria [15]. The low social support at work was assessed using the 26 items of the French version of the Karasek Job Content Questionnaire (JCQ) [18], and dichotomized using the median values of the French national SUMER (medical surveillance of occupational risk exposures) study to classify exposed and unexposed workers [19]. In addition, two combined factors were created. These were “high perceived physical exertion + low social support” (HPPELSS), which was categorized into four groups: no factor, low social support only, high physical exertion only, and both factors (high physical exertion and low social support); and “posture with arms above shoulder level + low social support” (PAASLSS), which was categorized into four groups: no factor, low social support only, posture with arms above shoulder level only, and both factors (posture with arms above shoulder level and low social support).

### 2.3. Statistical Analysis

To identify a hypothetical prevention model that would prevent more UEMSDs from occurring by addressing the modifiable occupational risk factor(s) with the greatest impact on the working population, two multivariate models, each including one of the two combinations of factors mentioned above, were tested, as described below.

(1) Model 1: HPPELSS + posture with arms above shoulder level + female sex + age.
(2) Model 2: PAASLSS + high perceived physical exertion + female sex + age.

As in the previous study [13], relative risks (RRs), PAFs, and population estimated numbers (PEN) of UEMSD cases attributable to risk factors were computed for each model. Briefly, PAFs were estimated using the method described by Spiegelman et al. [20] with the SAS (Statistical Analysis System) macro. The total number of incident UEMSDs in the working population of the PdL region was estimated after adjustment of the sample weights using the 2007 PdL region census data (see reference [13] for the estimation procedure previously described). The population estimated number of potentially preventable cases of UEMSD was calculated by multiplying PAFs by the total number of incident UEMSDs in the working population of the PdL region. To facilitate the comprehension and interpretation of the PAF estimate, the lower limit of its 95% confidence interval (CI) was set to zero when this lower limit was negative. All statistical analyses were performed using SAS software, version 9.4 TS Level 1M6.

## 3. Results

Of the 1246 workers (59% men, mean age: 38 years ± 8.6 at baseline), 139 (11.2%) developed a UEMSD during follow-up, amounting to a projected number of 129,320 new UEMSD cases in the PdL region working population in 2007 (Table 1). No significant difference of the incidence proportion of UEMSDs between sexes was observed in the working population of the PdL region (10.3% for men versus 12.4% for women; *p* = 0.287). The most common diagnoses at follow-up were RCS (incidence proportion 6.5%), LET (incidence proportion 2.2%), and CTS (incidence proportion 2.0%). Table 2 gives the RR for incident UEMSDs, PAFs, and population estimated numbers (PEN) of UEMSD cases attributable to risk factors, with the lowest risk group as a reference. In model 1, of the 129,320 new UEMSD cases estimated in the PdL region in 2007, low social support at work only led to an estimate of 8664 new cases representing 6.7% of all new cases, high perceived physical exertion at work only led to 19,010 (14.7%), and the combination of both factors led to 20,433 (15.8%). In model 2, 16,294 new cases (12.6% of all new cases in the PdL region) were attributable to low social support at work only, 6983 (5.4%) to working posture with arms above shoulder level at work only, and only 5043 (3.9%) were attributable to the combination of both factors.

## 4. Discussion

This cohort study analyzed two work-related UEMSD prevention models and supports the need for prevention programs to adopt a multidimensional approach that aims at reducing both biomechanical (particularly for high physical exertion) and psychosocial factors, as this would potentially prevent the occurrence of a larger number of UEMSD cases [21].

The reduction of exposure to high occupational physical exertion in combination with the improvement of social support at work may theoretically prevent 20,443 new cases, i.e., 16% of the 129,320 UEMSD cases projected in the PdL region in 2007 among workers. To the best of our knowledge, this is the first study estimating the number of cases that could potentially be prevented by multidimensional interventions acting on both biomechanical (i.e., high physical exertion) and psychosocial (i.e., low social support) exposure. Several studies have estimated the combined effect of biomechanical factors on the occurrence of UEMSDs [14,22,23,24,25], but only one study [26] has evaluated the combined effect of biomechanical and psychosocial factors. The study showed an increased risk of neck/shoulder symptoms with the combination of awkward or tiring position + awkward grip or hand movements + work stress. However, PAFs and the number of UEMSD cases attributable to the risk factors were not evaluated [26].

In the model of reducing exposure to working postures with arms above the shoulders combined with improved social support, approximately 5000 new UEMSD cases (3.9% of all new UEMSDs) could theoretically be avoided. The lower number of potentially preventable cases when acting on these two risk factors can be explained by the low proportion of workers exposed to the combination of both risk factors (joint prevalence), and the low number of UEMSD cases corresponding to the combination of both risk factors among these exposed workers. Indeed, these two key indicators (prevalence and number of UEMSD cases) that are taken into account in the PAF calculation [27] are lower among workers exposed to both factors than among those exposed to only one of these risk factors.

The main strength of this study was the use of a prospective cohort which included a representative sample of the working population at baseline. Furthermore, information on exposures was collected based on literature definitions or public health recommendations [15,17]. In addition, outcomes were clinically assessed by trained OPs using standardized procedures [15].

This study examined the potential impact of two multidimensional preventive intervention strategies on UEMSD cases prevented. One strategy acted on occupational exposure to both high physical exertion and low social support, and the other acted on working posture with arms above the shoulders and low social support. The low prevalence of certain risk factors (e.g., working with arms above the shoulders, low social support), their combination, or the low number of UEMSD cases led to low PAFs and therefore low numbers of attributable cases in this study. This was particularly the case with the combination of reducing working with arms above the shoulders and improving social support, which would prevent fewer UEMSDs than if only one of the two factors was acted upon in this study. Furthermore, the sample size in this study did not enable analysis by type of UEMSD. Some of the six UEMSDs analyzed (e.g., rotator cuff syndrome) were more sensitive to certain risk factors (e.g., working with arms above shoulder level). Depending on pathology profiles, we would have been able to better predict the axes of prevention to be prioritized if we had had a cohort with a larger number of workers with the possibility of differentiating the “winning” prevention strategies according to the types of pathologies. Another limitation is the PAF computation, which may have been affected by the thresholds used to define exposure levels [27]. However, to minimize the risk of bias, the choice of exposure definitions was made based on the scientific literature and public health recommendations. Finally, the PAF estimate assumes a causal relationship between the risk factor and the UEMSD, and should therefore be interpreted with caution. Given the above-mentioned limitations, future research on estimating preventable UEMSDs attributable to work should be conducted in longitudinal studies with larger sample sizes in order to obtain a solid scientific basis upon which to confirm our conclusions and also to perform similar analyses in different occupations and industrial sectors with a high risk of UEMSD. Future studies should evaluate the effectiveness and cost-effectiveness of such interventions.

## 5. Conclusions

In conclusion, this study demonstrated the potential relevance of multidimensional preventive interventions simultaneously acting on several occupational factors. The findings suggest that an intervention that would both reduce exposure to high physical exertion and improve social support at work could potentially reduce the incidence of work-related UEMSDs, thereby preventing a large number of cases. These conclusions support the implementation of interventions that target a combination of occupational risk factors in order to maximize the number of potentially preventable cases, the effectiveness of which should be evaluated in future studies.

## Figures and Tables

**Table 1:** Distribution of the six upper-extremity musculoskeletal disorders (UEMSDs) among the study population and its projection at the level of the Pays de la Loire (PdL) region.

	Study Sample							Projection of the Study Sample at the Level of the PdL Region						
	Overall (N = 1246)		Men (N = 734)		Women (N = 512)		*p*	Overall (N = 1,141,324) ^¥^		Men (N = 582,950) ^¥^		Women (N = 558,373) ^¥^		*p* ^#^
	N	%	n	%	n	%		n	%	n	%	n	%	
Rotator cuff syndrome (RCS)	78	6.3	41	5.6	37	7.2	0.242	73,858	6.5	32,827	5.6	41,032	7.3	0.259
Lateral epicondylar tendinopathy (LET)	28	2.3	22	3.0	6	1.2	0.032	24,767	2.2	18,117	3.1	6650	1.2	0.033
Carpal tunnel syndrome (CTS)	24	1.9	7	1.0	17	3.3	0.003	22,456	2.0	7228	1.2	15,228	2.7	0.084
Ulnar tunnel syndrome	12	1.0	7	1.0	5	1.0	1.000 *	12,022	1.1	7796	1.3	4227	0.8	0.332
De Quervain tenosynovitis	10	0.8	4	0.6	6	1.2	0.334 *	7878	0.7	2159	0.4	5719	1.0	0.138
Flexor-extensor peritendinitis or tenosynovitis of the forearm-wrist region	9	0.7	5	0.7	4	0.8	1.000 *	9399	0.8	3988	0.7	5410	1.0	0.625
At least one of the six UEMSDs	139	11.2	74	10.1	65	12.7	0.149	129,320	11.3	60,133	10.3	69,187	12.4	0.287

*p*-value of chi-square test; * Fisher’s exact test; ^¥^ weighted; ^#^ *p*-value of the Rao–Scott chi-square test for weighted samples.

**Table 2:** Proportion and population estimated number (PEN) of incident UEMSDs attributable to exposure to occupational risk factors in the working population in the PdL region.

**Model 1**	**Estimates in the COSALI Cohort**			**Estimates at the Level of the PdL Region**
	Prev * (%)	**Adjusted RR (95% CI)**	**Adjusted PAF (95% CI)**	**PEN (Variation Range)**
Occupational factors				
HPPELSS: high perceived physical exertion (Borg RPE scale ≥13) + low social support				
No factor	37.2	1.00	Reference	Reference
Low social support only	17.0	1.64 (1.00 to 2.70)	6.7 (0.7 to 12.7)	8664 (905 to 16,424)
High perceived physical exertion only	27.2	1.83 (1.18 to 2.82)	14.7 (5.4 to 23.7)	19,010 (6983 to 30,649)
Both factors ^#^	18.6	2.37 (1.51 to 3.70)	15.8 (8.9 to 22.5)	20,433 (11,509 to 29,097)
Posture with arms above shoulder level (≥2 h/day)	10.1	1.72 (1.14 to 2.57)	7.5 (0.5 to 14.4)	9699 (647 to 18,622)
Personal factors				
Female sex	41.1	1.32 (0.97 to 1.80)	11.4 (0 to 23.8)	14,742 (0 to 30,778)
Age: 35–44 years	36.2	1.51 (1.01 to 2.25)	12.1 (1.5 to 22.5)	15,648 (1940 to 29,097)
Age: ≥45 years	26.9	2.09 (1.41 to 3.10)	19.5 (10.3 to 28.4)	25,217 (13,320 to 36,727)
**Model 2**	**Estimates in the COSALI cohort**			**Estimates at the level of the PdL region**
	Prev * (%)	**Adjusted RR (95% CI)**	**Adjusted PAF (95% CI)**	**PEN (variation range)**
Occupational factors				
PAASLSS: posture with arms above shoulder level + low social support				
No factor	58.4	1.00	Reference	Reference
Low social support only	31.5	1.51 (1.07 to 2.14)	12.6 (2.7 to 22.3)	16,294 (3492 to 28,838)
Posture with arms above shoulder level only	6.0	2.00 (1.18 to 3.37)	5.4 (0 to 10.8)	6983 (0 to 13,967)
Both factors ^$^	4.1	2.17 (1.17 to 4.03)	3.9 (0.3 to 7.4)	5043 (388 to 9570)
High perceived physical exertion (Borg RPE scale ≥13)	45.8	1.63 (1.17 to 2.28)	23.2 (6.0 to 39.0)	30,002 (7759 to 50,435)
Personal factors				
Female sex	41.1	1.32 (0.97 to 1.80)	11.4 (0 to 23.7)	14,742 (0 to 30,649)
Age: 35–44 years	36.2	1.50 (1.01 to 2.23)	12.0 (1.2 to 22.5)	15,518 (1552 to 29,097)
Age: ≥45 years	26.9	2.09 (1.41 to 3.09)	19.5 (10.4 to 28.3)	25,217 (13,449 to 36,598)

UEMSDs: upper-extremity musculoskeletal disorders; ^#^ high physical exertion and low social support; ^$^ posture with arms above shoulder level and low social support; * prevalence of risk factor; RR: relative risk; 95% CI: 95% confidence interval. The PAF, adjusted for all factors in the model, was calculated using the lowest risk group for each factor as the reference group, with all other factors remaining unchanged. PAF: population attributable fraction; PEN: population estimated number of UEMSDs. The PEN factor was calculated by multiplying the PAF by the projected number of incident UEMSD cases in the Pays de la Loire region in 2007.

## Data Availability

The datasets used and/or analyzed during the current study are available from the corresponding author or J.B. or Y.R. on reasonable request.
